# Postoperative Immobilization After Hip Reconstruction in Cerebral Palsy: No Difference Between Hip Spica and Abduction Pillow

**DOI:** 10.3389/fsurg.2022.863287

**Published:** 2022-06-06

**Authors:** Alexander L. Vasconcellos, Alex S. Tagawa, Jason T. Rhodes, Lori J. Silveira, Austin A. Skinner, David B. Frumberg

**Affiliations:** ^1^ Department of Orthopaedic Surgery, University of Minnesota School of Medicine, Minneapolis, MN, United States; ^2^ Center for Movement and Gait Analysis, Children’s Hospital Colorado, Anschutz Medical Campus, Aurora, CO, United States; ^3^ Department of Orthopaedic Surgery, University of Colorado School of Medicine, Aurora, CO, United States; ^4^ Department of Pediatrics, Children’s Hospital Colorado, Anschutz Medical Campus, Aurora, CO, United States; ^5^ Department of Orthopaedics and Rehabilitation, Yale University School of Medicine, New Haven, CT, United States

**Keywords:** abduction pillow, spica cast, immobilization, hip reconstruction, cerebral palsy

## Abstract

**Purpose:**

This study aims to compare radiographic outcomes and complication rates of immobilization with an abduction pillow to spica casting for postoperative care after a hip reconstruction with varus derotational proximal femur osteotomy (VDRO) with or without pelvic osteotomy for children with cerebral palsy (CP).

**Methods:**

233 children (1–18 years old) diagnosed with CP that underwent VDRO with or without pelvic osteotomy were identified, of which 188 patients were immobilized with a spica cast and 45 were immobilized with an abduction pillow, based on surgeon preference. 123 (65%) in the Spica group and 21 (47%) in the pillow group had pelvic osteotomies. Demographic data and complication rates were collected. Radiographic parameters, including anatomic medial proximal femoral angle (aMPFA), acetabular index (AI) and migration percentage (MP), were measured for each patient at the completion of surgery, six weeks post-operatively, and one year post-operatively.

**Results:**

There was not a statistically significant difference in BMI (*p* = 0.285), gender distribution (*p* = 0.984), or median follow-up time (*p* = 0.314) between groups. Rates of complications were consistent among groups with no differences in instances of delayed unions (*p* = 0.10), subluxations (*p* = 0.55), infection (*p* = 0.71), or non-unions (*p* = 0.10). There was no statistically significant difference in number of patients with an ideal aMPFA, AI, or MP (*p* = 0.44, *p* = 0.19, *p* = 1.00) at one year post-operatively.

**Conclusions:**

Immobilization with an abduction pillow is a safe and effective alternative to hip spica casting following hip reconstruction.

## Introduction

**Figure 1:**
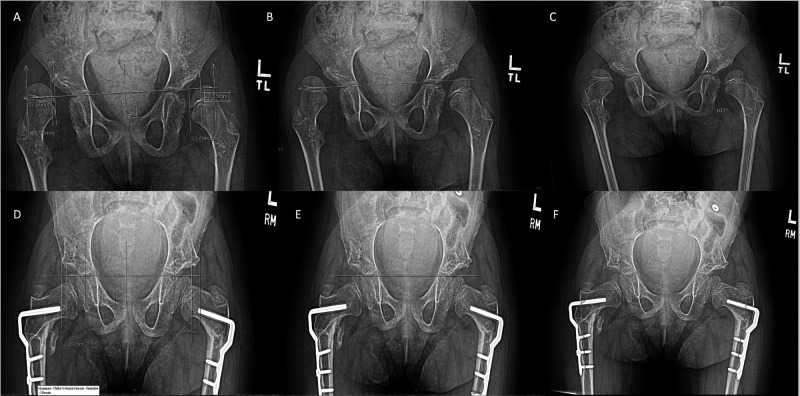
Measurements of a single patient pre- and post-operative of a VDRO for a single patient. (**A–C**) are pre-operative MP, AI, aMPFA respectively, while (**D–F**) are post-operative MP, AI, aMPFA respectively.

Cerebral palsy (CP) is a heterogeneous neurodevelopmental disorder which frequently results in neuromuscular hip dysplasia (also referred to as neurogenic dislocation of the hip) (1, 2). Commonly observed pathologies include coxa valga, increased femoral anteversion, and posterosuperior acetabular deficiency, which can lead to hip dislocation (3). Numerous guidelines and algorithms for periodic surveillance of the hips in CP patients exist, typically relying on clinical examination and an anteroposterior pelvic radiograph to track progression of these findings. Radiographically, the anatomical medial proximal femoral angle (aMPFA) (Figures 1C, F) can be used to measure the relative coronal plane alignment of the proximal femur (4, 5). The Reimers index/migration percentage (MP) (Figures 1A, D) and acetabular index (AI) (Figures 1B, E) can be measured to evaluate the location of the femoral head within the socket, and degree of acetabular dysplasia respectively (6, 7).

The risk of hip migration is directly correlated with increasing level Gross Motor Function Classification System (8), thus necessitating the need for more frequent surveillance with increasing severity of involvement (9).

Reconstructive hip surgery has been shown to be an effective means of treating symptomatic or unacceptably aligned hips (10, 11). This typically consists of a varus derotation osteotomy of the proximal femur (VDRO) to correct coxa valga and excess anteversion, and/or pelvic osteotomy for correction of acetabular dysplasia (12). While in the absence of pelvic osteotomies one may not expect there be improvement in AI, there is evidence that a VDRO alone may instigate bony remodeling of the acetabulum (3, 12, 13).

Absolute indications for VDRO include an MP greater than 50% or new hip dislocations with a break in Shenton’s line. Relative indications include a rapid increase in MP, MP greater than 40% with hip pain, difficulty with perineal hygiene, gait dysfunction and seating intolerance (10, 14, 15). An absolute indication for addition of a pelvic osteotomy is an acetabular index (AI) greater than 30 degrees with associated instability (16), with relative indications for an AI greater than 30 degrees, a level 5 GMFCS, or if the patient is older than 6 years of age (10, 12, 17, 18).

**Figure 2:**
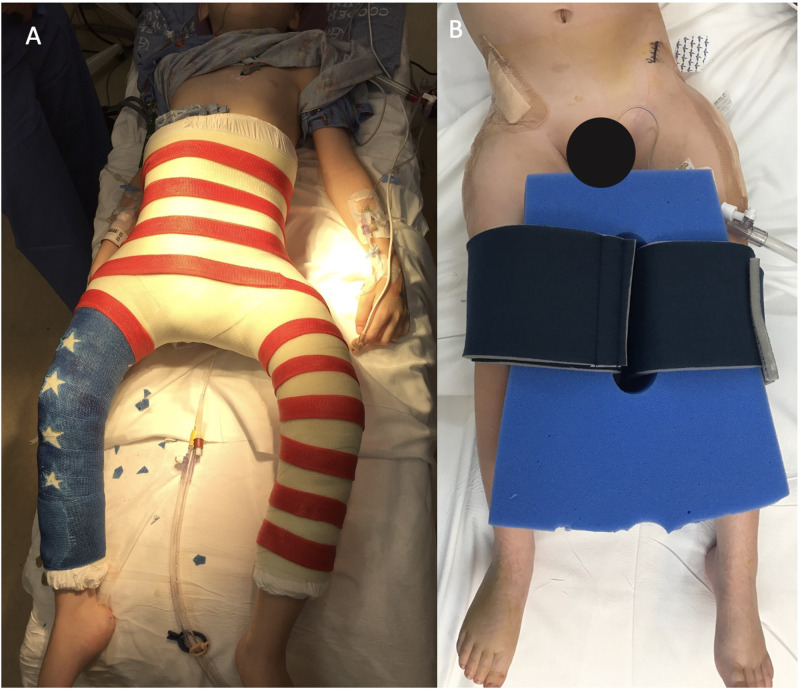
Immobilization after a Varus Derotational Osteotomy. (**A**) shows an example of a spica cast. (**B**) shows an example of an abduction wedge pillow.

Historically, post-operative immobilization has most commonly included hip spica casting (Figure 2A) (11, 15, 19). Theoretical advantages of casting include stability of the bony reconstruction prior to bony union, maintenance of articular alignment, and protection of incisions, and as such some surgeons utilize them in patients with poor bone quality or if there is concern for the fixation achieved intraoperatively (17, 19–21). There are known postoperative complications when using hip spica casts, including skin sores, hygiene difficulties, need for specialized car seats, wheelchair challenges, prolonged anesthesia time, and psychosocial impacts on both the patient and their family (22). Casting may be unnecessary due to the stability of contemporary internal fixation hardware used for proximal femur osteotomies, as well as the stability of commonly used pelvic osteotomy techniques (23).

Postoperative care strategies are highly variable between surgeons and institutions, with large differences in immobilization strategies, and this lack of consensus is in part due to the lack of substantial evidence directly comparing different methods of immobilization. Described alternatives have included long leg splints with abduction wedge, abduction wedge alone, Petrie cast, long leg splints alone, night splint foam, short leg casts with derotation bar and knee immobilizers, hip abduction orthosis, or no immobilization at all (19). The most common method outside spica casting at our institution is an abduction pillow, a commonly utilized method according to a recent survey by Miller et al. (19) (Figure 2B). Length of immobilization was 6 weeks in spica cast, and 6 weeks in abduction pillow.

This study characterizes the radiographic outcomes and postoperative complication profile between two methods of post-operative immobilization utilized at our institution after hip reconstruction for patients with CP. We theorize that radiographic outcomes and rates of postoperative complication in patients treated with abduction pillows are not significantly different than hip spica casts. Abduction pillows may obviate the challenges that hip spica casts pose to care teams, patients and their parents in the post-operative period.

## Materials and Methods

### Subjects

After approval from our institution’s ethical review board, a retrospective cohort study was created, in which patient consent was not required. We examined a consecutive cohort of children 1–18 years of age with CP that underwent unilateral or bilateral VDRO with or without pelvic osteotomy (San Diego osteotomy) at a single institution between 1999 and 2017. Patients undergoing single event multi-level surgery (SEMLS) were not included. Only patients that had clearly documented immobilization with a hip spica cast or abduction pillow post-operatively were included. Patients were excluded if they did not have one year of follow-up, or if they had a concomitant diagnosis that might significantly change their course of recovery (e.g., collagen-vascular disorder). 436 hip reconstructions in 233 patients were included in the analysis. 188 patients (357 hips) were immobilized in a hip spica cast post-operatively, while 45 patients (79 hips) were immobilized with an abduction pillow with or without knee immobilizers. For the purposes of comparing radiographic outcomes, number of hips operated on was used, while post-operative complications are compared on an individual patient basis.

### Outcome Variables

Demographic data and complications were reviewed for the post-operative period. All immobilization was maintained for at least 6 weeks post-operatively, with eventual weaning by 3 months post-operatively. AP pelvis radiographs, as well as intra-operative fluoroscopic images, were used for radiographic assessment. aMPFA was measured at index using intraoperative fluoroscopy. AI, MP, and aMPFA were measured at six weeks post-operatively and one year post-operatively. AI and MP could not be accurately assessed via index fluoroscopy as they require an image with both hips in view.

### Statistical Methods

Descriptive statistics including mean and standard deviation were used to characterize continuous variables. Paired t-tests were used to evaluate demographic differences between the immobilization groups. Ideal radiographic measurements were defined as follows: aMPFA = 80–88°, AI = 20–26°, MP less than 15% (2), the number of patients meeting these parameters in each group was documented for each measured time point. Fisher’s exact test was used to evaluate difference between the two groups. Change in each radiographic parameter over follow-up time was also analyzed and compared between the two groups.

## Results

Demographic data and disease characteristics are summarized in Tables 1 and 2, demonstrating the overall similarity of the two groups. All patients underwent at least a VDRO. 21 (26.9% of patients treated with abduction pillow underwent pelvic osteotomy in addition to VDRO, compared to 123 (34.5%) treated with spica cast (*p* = 0.23). There were no differences between the spica cast and the abduction pillow group for sex distribution, ethnicity, BMI, follow up time. The abduction pillow group was significantly older, taller, and heavier on average. There was no significant difference in GMFCS classification or CP type between the two groups. There was a difference in CP diagnosis with more quadriplegic and triplegic involvement in the casting group.

**Table 1:** Demographics.

	Abduction Pillow *n* = 45 (79 hips)	Spica Cast *n* = 188 (379 hips)	*p*-value
Number with pelvic osteotomy (%)			0.23
	21 (26.9%)	123 (34.5%)	
Sex			0.984
Male (%)	23 (51.1)	99 (52.7)	
Female (%)	22 (48.9)	89 (47.3)	
Age at Surgery in years (Median [IQR])			0.004*
	7.53 [5.04, 13.57]	6.06 [4.21, 8.39]	
Follow Up Time in years (Median [IQR])			0.314
	0.90 [0.66, 1.15]	0.92 [0.74, 1.23]	
Height in cm (Median [IQR])			<0.001*
	115.00 [102.00, 142.00]	104.00 [94.00, 117.00]	
Weight in kg (Median [IQR])			<0.001*
	21.50 [16.10, 36.20]	16.80 [13.80, 21.00]	
BMI (Median [IQR])			0.285
	16.03 [14.08, 18.17]	15.18 [14.07, 17.35]	
Ethnicity			0.689
Hispanic (%)	14 (31.1)	57 (30.3)	
Caucasian (%)	23 (51.1)	95 (50.5)	
African American (%)	2 (4.4)	15 (8.0)	
American Indian/Alaska Native (%)	0 (0.0)	4 (2.1)	
Asian (%)	0 (0.0)	1 (0.5)	
Mixed (%)	4 (8.9)	7 (3.7)	
Not Reported (%)	2 (4.4)	6 (3.2)	
Other (%)	0 (0.0)	3 (1.6)	

* *Statistically significant (p < 0.05).*

**Table 2:** Disease characteristics.

	Abduction Pillow *n* = 45 (79 hips)	Spica Cast *n* = 188 (379 hips)	*p*-value
GMFCS			0.237
Unknown/Not reported (%)	0 (0.0)	9 (4.8)	
Level I (%)	2 (4.4)	4 (2.1)	
Level II (%)	0 (0.0)	7 (3.7)	
Level III (%)	4 (8.9)	14 (7.4)	
Level IV (%)	11 (24.4)	28 (14.9)	
Level V (%)	28 (62.2)	126 (67.0)	
CP Diagnosis			0.040*
Hemiplegic (%)	2 (4.4)	8 (4.3)	
Diplegia (%)	12 (26.7)	29 (15.4)	
Triplegic (%)	0 (0.0)	7 (3.7)	
Quadriplegic (%)	30 (66.7)	141 (75.0)	
Not specified (%)	1 (2.2)	3 (1.6)	
Type of CP			0.63
Spastic (%)	27 (34.2)	125 (35.0)	
Dystonic/athetoid (%)	0 (0.0)	3 (0.8)	
Spastic with dystonia (%)	46 (58.2)	213 (59.7)	
Hypotonic (%)	6 (7.6)	14 (3.9)	
Ataxic (%)	0 (0.0)	0 (0.0)	
Unspecified (%)	0 (0.0)	2 (0.6)	

* *Statistically significant (p < 0.05).*

### Radiographic Outcomes

#### aMPFA (Table 3)

Mean measurements were similar throughout the course of follow up in both groups (Table 3). There was no statistically significant difference in number of hips with ideal measurements at any time point, and no difference in angular change over the year of follow up.

**Table 3:** Anatomical medial proximal femoral angle.

	Abduction pillow *n* = 45 (79 hips)	Spica cast *n* = 188 (379 hips)	*p*-value
Mean in degrees (SD) at each time point			
Index	84.6 (10.1)	85.6 (9.7)	
6 week follow up	84.0 (9.7)	85.8 (11.0)	
1 year follow up	85.6 (11.7)	85.5 (12.5)	
Number (% of total) in ideal range (80–88 degrees)			
Index	29 (36.7%)	117 (33.7%)	0.6
6 week follow up	21 (31.3%)	87 (35.1%)	0.66
1 year follow up	15 (24.2%)	87 (30.0%)	0.44
Differences in aMPFA in degrees (SD) over time			
Index to 6 week follow up	−.85 (4.6)	−0.61 (7.2)	0.75
6 week to 1 year follow up	0.45 (5.3)	−0.40 (7.6)	0.34
Index to 1 year follow up	0 (5.7)	−0.84 (8.3)	0.34

* *Statistically significant (p < 0.05).*

#### AI (Table 4)

Mean measurements were also similar throughout the course of follow up, and there was never a statistically significant difference in number of hips with ideal measurements (Table 4). There was a higher percentage of hips treated with spica cast with improved AI at index, however this difference was not significant and decreased over the year of follow up. There was no difference in change in AI over the course of follow up between the two groups.

**Table 4:** Acetabular index.

	Abduction pillow *n* = 45 (79 hips)	Spica cast *n* = 188 (379 hips)	*p*-value
Mean in degrees (SD) at each time point			
Index to 1 year follow up	–	–	–
6 week follow up	21.5 (6.2)	22.3 (7.3)	–
1 year follow up	21.3 (5.8)	21.7 (6.8)	–
Number (% of total) in ideal range (20–26 degrees)			
Index	–	–	–
6 week follow up	19 (29.2%)	135 (40.7%)	0.1
1 year follow up	28 (48.3%)	121 (38.2%)	0.19
Differences in AI in degrees (SD) over time			
Index to 6 week follow up	–	–	–
6 week to 1 year follow up	−0.29 (3.9)	−0.89 (4.9)	0.31
Index to 1 year follow up	–	–	–

* *Statistically significant (p < 0.05).*

#### MP (Table 5)

MP was also similar at all time points between the two groups (Table 5). There was a statistically significant difference at 6 week follow up in the number of patients with ideal measurements (*p* = 0.0021), however in both groups by the 1 year follow up 100% ideal measurements were obtained. There was a statistically significant change in MP over time between the two groups with a change of 4% in the pillow group and 7% in the spica group (*p* = 0.02).

**Table 5:** Migration percent.

	Abduction pillow *n* = 45 (79 hips)	Spica cast *n* = 188 (379 hips)	*p*-value
Mean in percent (SD) at each time point			
Index to 1 year follow up	–	–	–
6 week follow up	8% (10%)	6% (13%)	–
1 year follow up	12% (12%)	13% (17%)	–
Number (% of total) in ideal range (<15 percent)			
Index	–	–	–
6 week follow up	53 (69.7%)	280 (85.6%)	0.0021*
1 year follow up	67 (100%)	319 (100%)	1
Differences in MP in percent (SD) over time			
Index to 6 week follow up	–	–	–
6 week to 1 year follow up	4% (7%)	7% (12%)	0.02*
Index to 1 year follow up	–	–	–

*Statistically significant (p < 0.05).

### Postoperative Complications

Documented postoperative complications are detailed in Table 6. There were no significant differences between the two groups in rates of any complication.

**Table 6:** Complications.

	Abduction Pillow *n* = 45	Spica Cast *n* = 188	*p*-value
Presence of Delayed Union	1 (2.2%)	4 (2.1%)	0.1
Presence of Subluxation	2 (4.4%)	17 (9.4%)	0.55
Presence of Non-Union	1 (2.2%)	4 (2.1%)	0.1
Avascular Necrosis within 1 year	0 (0.0%)	1 (0.5%)	1
Postop fever	2 (4.4%)	20 (10.6%)	0.39
Pressure Sores	6 (13.3%)	10 (5.3%)	0.051
Patient was Unable to Tolerate Immobilization	0 (0.0%)	1 (0.5%)	1
Infection related complications			
Pneumonia	1 (2.2%)	0 (0%)	0.18
Urinary Tract Infection	1 (2.2%)	2 (1.1%	0.45
Incision Wound Infection	1 (2.2%)	7 (3.7%	0.36
Infection Types Overall	3 (6.7%)	9 (4.8%)	0.71
Incidence of all infection related complications	6.67 per 100	4.79 per 100	–

* *Statistically significant (p < 0.05).*

## Discussion

While hip spica casts were classically considered the standard for immobilization following hip reconstructive surgery in children with CP, there is evidence that satisfactory outcomes can be obtained with alternative methods of postoperative immobilization (19, 24, 25). Spica casts have been actively avoided by families and care teams familiar with them because they are cumbersome and associated with other challenges during care (26). Albrektson et al. compared the two methods in a retrospective review of 21 patients with CP or other genetic syndromes undergoing hip surgery and concluded that spica casts should be used in patients with osteopenia or instability in the early post-operative period (21). Truong et al. demonstrated similar pain and complication profiles in children treated in spica casts versus short leg casts linked with a bar (27). Modern internal fixation has proven reliable in maintaining the alignment and compression of osteotomies during healing. The stability of pelvic osteotomy techniques, when used, has also allowed many surgeons to view spica casting as optional. Miller et al. recently showed that there is a tremendous lack of consensus in postoperative immobilization for CP hip reconstruction (19). This study sought to directly compare outcomes of hip reconstruction at a single institution in children that were immobilized with spica casts versus abduction pillows and better elucidate the complication rate of these methods.

This review included 436 VDROs in 233 patients at a single institution, with similar demographic data between the two immobilization groups. There was no difference in the distribution of GMFCS level, with GMFCS V being most common. There were more children that underwent concomitant pelvic osteotomies in the casting group, but this was not a significant difference. The group of patients immobilized in a cast were significantly younger, shorter, and weighed less than the spica cast group. This is likely due to the decreased feasibility of caring for a child in a spica cast with increasing patient size.

The method of post-operative immobilization did not significantly affect radiographic parameters at one year. There was no difference in femoral correction (aMPFA) or acetabular correction (AI) between the two groups throughout the follow-up period. MP was more likely to be in the ideal range at 6 weeks in hips immobilized with casts, but this difference was not significant between the groups at 1 year. It was found that the MP changed less over time in the abduction pillow compared to the spica cast group. This may be due to the overall younger age and increased number of pelvic osteotomies in the spica group, creating a cohort with more remodeling potential in the hip (28, 29). Hip reduction has been shown to reliably improve over time following hip reconstruction (2, 12, 13). Every hip in this series had a MP less than 15% at one year, thus this initial difference in the depth of hip reduction did not affect the overall success of reconstruction.

There was no difference in any complication between the two groups. Skeletal complications such as delayed union, nonunion, and surgical site infection were not significantly different, with only one case of avascular necrosis identified by one year in the cohort. Medical complications were also similar between both groups. A common belief is that spica casts can result in skin sores, and this was observed in 5% of patients immobilized in this manner. However, 13% of patients immobilized with abduction pillows had documented pressure sores as well, either at bony prominences (e.g., sacrum) or from the immobilizing device itself (e.g., strap of pillow over the knee). It is important for clinicians and families to be aware that neither method of immobilization is benign—careful attention to skin integrity is mandatory while the devices are in place, and our facility has developed a protocol to prevent these pressure sores with the abduction pillow.

Our retrospective study has some limitations. There were unequal numbers in each treatment group, reflecting the change in practice that occurred during the study period. This non-randomized study allowed surgeons to choose immobilization method based on preference, which introduces selection bias in assigning the method used and also fails to control for confounders (e.g., osteopenia). Both groups were very similar and represent an accurate snapshot of the broad distribution of children with CP that undergo hip reconstruction. As discussed, the spica group was younger and smaller, likely reflecting surgeon preference of avoiding casts in larger, older patients less likely to tolerate them. There is no established protocol regarding this at our institution. Patient positioning for pelvic radiographs is sometimes difficult at 6 weeks due to patient discomfort, especially from joint stiffness if they were just removed from a cast. This could result in inaccurate measurements at that time point, but there was ultimately a lack of statistical difference at 1 year in any of our measurements. Some patients were excluded due to other diagnoses that may have altered treatment plans, such as collagen or genetic disorders and other bone dysplasias. The surgeon and family should discuss how to immobilize these patients with whichever method is safest based on their individual needs. This study did not examine compliance with the wedge pillow, as it is theoretically removable. A major goal of employing a pillow instead of a cast is to decrease the burden on the family. This study lacks a qualitative measure of treatment-related quality of life that could better enable surgeons to understand the perspective of the families they treat, which could ultimately contribute to the choice of immobilization. This paper also did not address patients specifically undergoing multi-level surgery, in which other immobilization in the lower limbs may preclude utilization of a pillow. In these situations surgeon’s may consider using a pillow in conjunction with casts versus an alternative method. Reconstruction frequently alleviates contractures about the hip, but caution should be considered if there is any residual asymmetry (e.g., windswept deformity).

## Conclusion

The use of a spica casts after hip reconstruction in CP presents many challenges to the patient, family, and clinician. This study sought to compare outcomes of hip reconstruction after which children were immobilized with spica casts or hip abduction pillows. There were no differences in osteotomy alignment or healing, and ultimate hip reduction was excellent regardless of which method was used. Skin complications were observed in both groups. This would suggest it is reasonable to utilize a patient focused shared decision making conversation as part of the process in determining what type of immobilization would be best. Future research is needed to determine if the elimination of spica casting does truly lessen the treatment burden on families while still ensuring safe immobilization.

## Data Availability

The raw data supporting the conclusions of this article will be made available by the authors, without undue reservation.
